# The relationship between insomnia symptoms and work productivity among blue-collar and white-collar Japanese workers engaged in construction/civil engineering work: a cross-sectional study

**DOI:** 10.1186/s12889-021-11273-y

**Published:** 2021-06-28

**Authors:** Momoko Kayaba, Taeko Sasai-Sakuma, Yoshikazu Takaesu, Yuichi Inoue

**Affiliations:** 1 Department of Somnology, Tokyo Medical University, 6-7-1 Nishishinjuku, Shinjuku-ku, Tokyo, 160-0023 Japan; 2 Japan Somnology Center, Institute of Neuropsychiatry, 5-10-10, Yoyogi, Shibuya-ku, Tokyo, 151-0053 Japan; 3 Department of Clinical Laboratory Science, Faculty of Medical Technology, Teikyo University, Kaga 2-11-1, Itabashi-ku, Tokyo, 173-8605 Japan; 4 Department of Neuropsychiatry, Kyorin University School of Medicine, Shinkawa 6-20-2, Mitaka-shi, Tokyo, 181-8611 Japan; 5 Department of Neuropsychiatry, Graduate School of Medicine, University of the Ryukyus, 207 Uehara, Nishihara, Okinawa, 903-0215 Japan

**Keywords:** Work productivity, Work performance, Absenteeism, Insomnia, Blue-collar worker, Shift work

## Abstract

**Background:**

The situation of work productivity loss due to sleep disorders/problems among workers in industrialized societies remains unclear. The purpose of this study was to clarify the prevalence of insomnia symptoms and actual situation of work productivity by job type (white-collars/blue-collars) among construction/civil engineering workers in Japan and evaluate the association between insomnia symptoms and work productivity adjusting for sleep duration and sociodemographic, work-related, and health-related variables.

**Methods:**

This cross-sectional study included 17,828 construction/civil engineering workers (15,837 males and 1991 females) aged 40 to 74 years in Japan. The questionnaire consisted of socio-demographic characteristics, information on work productivity (work performance and absence), respective insomnia symptoms (difficulty initiating sleep; DIS, difficulty maintaining sleep; DMS, and early morning awakening; EMA), bedtime schedule, work-related factors (job type, working hours), and perceived health condition. To identify the associated factors of work productivity, the logistic regression analyses were conducted.

**Results:**

The percentages of workers who reported to be experiencing DIS, DMS, and EMA were 7.9, 16.3, and 13.1%, respectively. Poor work performance was associated with every insomnia symptom in both the blue-collar and white-collar workers. Meanwhile, absence was associated with DIS in blue-collar workers and both DIS and DMS in white-collar workers; however, not with EMA in both the groups. In blue-collar workers, engagement in shift work was associated with poor work performance.

**Conclusions:**

The present study revealed the association between insomnia symptoms and work productivity, suggesting the necessity of early prevention of insomnia among both blue-collar and white-collar workers.

**Supplementary Information:**

The online version contains supplementary material available at 10.1186/s12889-021-11273-y.

## Background

Loss of work productivity due to presenteeism, namely, the worker although being at work is less productive because of health problems, has recently drawn social attention [1]. The conditions causing loss of work productivity include various diseases such as, migraine, allergic rhinitis, depression, low back pain, and rheumatoid arthritis [2, 3]. Sleep disturbance also impairs daytime function, possibly leading to the loss of work productivity. Espie et al. reported that weekly frequency of symptom occurrence, morbidity duration, symptom constituents including difficulty falling asleep, and early morning awakening were significantly associated with presenteeism among workers of a global manufacturing company [4]. In addition, they showed that the levels of presenteeism varied depending on job type (office workers < retail; service workers < plant workers) and gender (male and female). A study using the United States (US) poll data also reported that presenteeism and absenteeism were associated with insomnia, obstructive sleep apnea, and restless legs syndrome; however, not with shift workers and shift work disorders [5]. A nationwide study confirmed the association between insomnia and both presenteeism and absenteeism after adjusting for sociodemographic characteristics and health status [6]. Another US nationwide study reported association of insomnia with presenteeism; however, not with absenteeism after other comorbid conditions were controlled [7].

Only a few studies have investigated the association between these problems and work productivity after adjustment of work-related factors (e.g., job type, work hours, shift work), which may be the confounders [4, 8]. According to previous studies on the relationship between work-related factors and workers’ health, psychological distress was more prevalent among blue-collar workers [9, 10] and shift workers [11]. Hilton et al. reported that productivity decrement due to psychological distress was higher in the blue-collar workers (25%) than in white-collar workers (6%) [12]. Considering this, this study was conducted: 1) to clarify the prevalence of insomnia symptoms and the actual situation of work productivity by job type (i.e., white-collars/blue-collars) among construction/civil engineering workers in Japan, and 2) to evaluate the association between insomnia symptoms and work productivity after adjustment of sleep duration and sociodemographic, work-related (i.e., shift work and working hours), and health-related variables.

## Methods

### Study design and population

Ethical approval for this study was obtained from the Ethical Committee of Japan Depression Center (No.2019_0002). This manuscript was written in accordance with the STROBE statement for cross-sectional studies and the checklist was cited as Additional file 1.

As a part of the health management of employees, the questionnaire was distributed to workers belonging to the health insurance society of construction/civil engineering in Japan. The present study analyzed the data of workers who gave informed consent for the questionnaire use for research purposes. Between April 2018 and March 2019, 26,773 workers aged 40 to 74 years, who received an annual specified medical checkup, responded to the questionnaire. Out of these, 8945 respondents were excluded according to the following exclusion criteria: lack of information whether living alone or not (*n* = 229), work performance (*n* = 541), number of days of absence in the previous 4 weeks (*n* = 1270), job type (*n* = 1925), working pattern (*n* = 3464), number of working days per week (*n* = 264) or working hours per week (*n* = 116), perceived physical health condition (*n* = 89), perceived mental health condition (*n* = 48), bedtime and wake-up time on weekdays and on weekends (*n* = 773), and presence/absence of subjective insomnia (*n* = 152). Finally, the completed data of 17,828 workers were analyzed for the present study.

### Measures

Among the socio-demographic variables, data on age and sex were collected by the insurance register. Other variables such as whether they were living alone or not, presence or absence of respective insomnia symptoms (difficulty initiating sleep; DIS, difficulty maintaining sleep; DMS, and early morning awakening; EMA), bedtime and wake-up time on weekdays and on weekends, job type (white-collars: designer, sales, clerical staff, or manager/blue-collars: field worker or overseer), working pattern (daytime work/shift work), number of working days per week, working hours per week (shorter than 40 h/40–60 h/60 h or longer), and perceived physical and mental health conditions (poor: very poor or poor/not poor: average, good, or very good) were self-reported or through rated questionnaire. Sleep duration on weekdays and weekends were calculated based on participants’ bedtime and wake-up time, following which that on weekdays was categorized into three groups (shorter than 7 h /7–8 h/8 h or longer) for subsequent analyses considering a U-shaped relationship (see Background). The work performance in the previous 4 weeks was assessed on a scale of 0% (“total lack of performance”) to 100% (“no lack of performance”) in units of 10%, while the absence was measured by asking the number of days an employee was absent for illness or injury in the same duration by using question items from the World Health Organization Health and Work Performance Questionnaire [13]. Work performance was converted into a binary outcome based on the median value. Absence was defined as the absence of more than a day in the previous 4 weeks.

### Statistical analysis

Representative values are shown as mean ± standard deviation. For comparisons between the groups of blue-collar and white-collar workers, the Wilcoxon’s rank sum test for continuous variables and Pearson’s Chi-squared test for categorical variables were used. To identify the associated factors of work productivity in white-collar and blue-collar, sub-group analyses using a logistic regression model were performed in which the response variables were work performance (90–100%/< 90%) and absence, (None/≥1 day) and explanatory variables were presence of DIS, DMS, and EMA, adjusting for age, sex, and whether the workers lived alone, sleep duration on weekdays (less than 7 h, 7–8 h, 8 h or longer), perceived physical health condition (good or average/poor), perceived mental health condition (good or average/poor), working pattern (daytime work/shift work), and hours engaged in work per week (shorter than 40 h /40–60 h/60 h or longer). In the logistic regression model, the degree of association was represented as adjusted odds ratio (aOR) and 95% confidence interval (95%CI). Statistical significance was set at *p* < 0.05. Statistical analyses were performed using R statistical software version 3.5.1 (R Core Team, Vienna, Austria), “caret” package [14], and “Resource Selection” package [15].

## Results

### Worker subjects’ characteristics

Sociodemographic, work-related, and health-related variables as well as sleep-related variables among the subject workers are shown in Table 1. The mean age was 52.5 ± 7.6 years (range: 40–74 years). A clear male predominance (88.8%) was observed, and 23.2% of the participants lived alone. About half of the workers were categorized as blue collar (40.9%), and 4.7% of the workers were engaged in shift work. The mean working days per week were 5.2 ± 0.5 days, with 17.2% of the workers working for less 40 h, 70.0% between 40 and 60 h, and 12.8% workers for 60 h or longer. The poor physical health was perceived in 12.5% of the workers, while 11.3% had poor mental health. The workers slept for an average of 6.3 ± 1.0 h on weekdays and 7.6 ± 1.2 h on weekends. There were 23.4% workers with sleep duration shorter than 7 h on weekdays, 69.5% had between 7 and 8 h, and 7.1% had 8 h or longer sleep duration.

**Table 1:** Subjects’ characteristics, insomnia symptoms and work productivity

		Total (*n* = 17,828)	Blue-collar (*n* = 7292)	White-collar (*n* = 10,536)	*p-*value
Age	(y)	52.5 ± 7.6	51.6 ± 7.8	53.2 ± 7.4	< 0.001^**^
Sex					
Male	n (%)	15,837 (88.8)	7175 (98.4)	8662 (82.2)	< 0.001^**^
Female	n (%)	1991 (11.2)	117 (1.6)	1874 (17.8)	
Living alone					
No	n (%)	4133 (23.2)	5181 (71.1)	8514 (80.8)	< 0.001^**^
Yes	n (%)	13,695 (76.8)	2111 (28.9)	2022 (19.2)	
Working pattern					
Daytime worker	n (%)	16,711 (93.7)	6452 (88.5)	10,259 (97.4)	< 0.001^**^
Shift worker	n (%)	834 (4.7)	795 (10.9)	39 (0.4)	
Working time					
40-60 h	n (%)	12,470 (69.9)	4861 (66.7)	7609 (72.2)	< 0.001**
< 40 h	n (%)	3068 (17.2)	783 (10.7)	2285 (21.7)	
≥ 60 h	n (%)	2290 (12.8)	1648 (22.6)	642 (6.1)	
Working days	(day/week)	5.2 ± 0.5	5.5 ± 0.6	5.1 ± 0.3	< 0.001^**^
Physical health					
Good/average	n (%)	15,603 (87.5)	6371 (87.4)	9232 (87.6)	0.631
Poor	n (%)	2225 (12.5)	921 (12.6)	1304 (12.4)	
Mental health					
Good/average	n (%)	15,807 (88.7)	6393 (87.7)	9414 (89.4)	< 0.01^*^
Poor	n (%)	2021 (11.3)	899 (12.3)	1122 (10.6)	
Sleep duration					
7-8 h	n (%)	4179 (23.4)	795 (24.2)	2415 (22.9)	< 0.001^**^
< 7 h	n (%)	12,385 (69.5)	4884 (67.0)	7501 (71.2)	
≥ 8 h	n (%)	1264 (7.1)	644 (8.8)	620 (5.9)	
Insomnia symptoms					
DIS	n (%)	1410 (7.9)	568 (7.8)	842 (8.0)	0.643
DMS	n (%)	2914 (16.3)	1166 (16.0)	1748 (16.6)	0.296
EMA	n (%)	2335 (13.1)	928 (12.7)	1407 (13.4)	0.230
Work performance					
90–100%	n (%)	8999 (50.5)	3198 (43.9)	5801 (55.1)	< 0.001^**^
< 90%	n (%)	8829 (49.5)	4094 (56.1)	4735 (44.9)	
Absence					
None	n (%)	16,163 (90.7)	6690 (91.7)	9473 (89.9)	< 0.001^**^
≥ 1 day	n (%)	1665 (9.3)	602 (8.3)	1063 (10.1)	

Values are expressed as mean ± standard deviation for continuous variables

*DIS* Difficulty initiating sleep, *DMS* Difficulty maintaining sleep, *EMA* Early morning awakening

* < 0.05, ** < 0.001

### Prevalence of insomnia symptoms

The percentages of workers who reported to be experiencing DIS, DMS, and EMA were 7.9, 16.3, and 13.1%, respectively. In all, 72.7% of workers reported no insomnia symptoms. Compared to the blue-collar workers, age (*p* < 0.001) and the percentage of female workers (*p* < 0.001) were higher, the percentages of workers who lived alone (*p* < 0.001) and those who were engaged in shift work (*p* < 0.001) were lower, working days (*p* < 0.001), working time (*p* < 0.001), and sleep duration (*p* < 0.001) were shorter, mental health (*p* < 0.01) and work performance (*p* < 0.001) were better, while absence(*p* < 0.001) was more prevalent among white-collar workers. Despite these significant differences, the prevalence of insomnia symptoms did not differ between the blue-collar and white-collar workers (*p* > 0.2).

### Work productivity

The median work performance was 90%. The percentage of workers who were absent due to sickness or injury for the previous 4 weeks was 9.3%. The mean number of absent days for the absentee workers during the study period was 3.0 ± 3.9 days.

The percentage of workers who had poor work performance was significantly higher in blue-collar workers than that in white-collar workers (56.1% > 44.9%, *p* < 0.001) (Table 1). On the other hand, the percentage of workers with positivity for absence was significantly higher in white-collar workers than in blue-collar workers (10.1% > 8.3%, p < 0.001) (Table 1).

### Relationship between insomnia symptoms and work productivity

The results of multiple logistic regression analyses of factors associated with subjectively poor work performance and absences among white-collar and blue-collar workers are outlined in Tables 2 and 3. Poor work performance was positively associated with insomnia symptoms (DIS, DMS, and EMA) among both blue-collar and white-collar workers. Absence was associated with DIS among both blue- and white-collar workers. DMS was only associated with absence in white-collar workers. EMA was not associated with absence in either blue- or white-collar workers.

**Table 2:** Logistic regression model for associated factors with poor work performance

	Blue-collar (*n* = 7292)		White-collar (*n* = 10,536)	
	Ref. 90–100% (*n* = 3198) / < 90% (*n* = 4094)		Ref. 90–100% (*n* = 4735) / < 90% (*n* = 5801)	
Explanatory variables	Crude OR (95% CI)	aOR^a^ (95% CI)	Crude OR (95% CI)	aOR^a^ (95% CI)
Age	0.98 (0.98–0.99)^**^	0.98 (0.98–0.99)^**^	0.99 (0.99–1.00)^*^	0.99 (0.99–1.00)^*^
Sex				
Male	1.0	1.0	1.0	1.0
Female	0.48 (0.33–0.70)^**^	0.43 (0.29–0.63)^**^	1.25 (1.13–1.38)^**^	1.10 (0.99–1.23)
Living alone				
No	1.0	1.0	1.0	1.0
Yes	1.29 (1.17–1.43)^**^	1.23 (1.11–1.37)^**^	1.13 (1.02–1.24)^*^	1.06 (0.96–1.17)
Working pattern				
Daytime worker	1.0	1.0	1.0	1.0
Shift worker	1.39 (1.21–1.60)^**^	1.40 (1.20–1.64)^**^	0.99 (0.87–1.13)	1.92 (1.00–3.80)
Working time				
40-60 h	1.0	1.0	1.0	1.0
< 40 h	0.98 (0.84–1.14)	1.05 (0.89–1.23)	1.30 (1.19–1.43)^**^	1.31 (1.18–1.45)^**^
≥ 60 h	1.39 (1.24–1.55)^**^	1.24 (1.10–1.41)	1.48 (1.26–1.74)^**^	1.40 (1.17–1.67)^**^
Physical health				
Good/average	1.0	1.0	1.0	1.0
Poor	2.89 (2.47–3.40)^**^	2.06 (1.73–2.47)^**^	2.52 (2.23–2.84)^**^	1.73 (1.52–1.98)^**^
Mental health				
Good/average	1.0	1.0	1.0	1.0
Poor	2.84 (2.42–3.34)^**^	1.82 (1.52–2.19)^**^	3.18 (2.78–3.64)^**^	2.23 (1.93–2.59)^**^
Sleep duration				
7-8 h	1.0	1.0	1.0	1.0
< 7 h	1.01 (0.91–1.13)	0.91 (0.81–1.02)	1.00 (0.91–1.10)	0.95 (0.86–1.05)
≥ 8 h	1.33 (1.11–1.60)^*^	1.41 (1.17–1.71)^**^	1.30 (1.09–1.55)^*^	1.26 (1.05–1.51)^*^
Insomnia symptoms				
Without DIS	1.0	1.0	1.0	1.0
Presence of DIS	1.74 (1.45–2.09)^**^	1.26 (1.04–1.53)^*^	1.80 (1.56–2.07)^**^	1.33 (1.14–1.55)^**^
Without DMS	1.0	1.0	1.0	1.0
Presence of DMS	1.66 (1.46–1.90)^**^	1.28 (1.11–1.48)^*^	1.62 (1.46–1.79)^**^	1.24 (1.11–1.39)^**^
Without EMA	1.0	1.0	1.0	1.0
Presence of EMA	1.56 (1.35–1.80)^**^	1.23 (1.05–1.44)^*^	1.67 (1.49–1.87)^**^	1.34 (1.19–1.52)^**^
Hosmer-Lemeshow goodness of fit test		*p* = 0.706		*p* = 0.350

*OR* Odds ratio, *CI* Confidence interval, *aOR* Adjusted odds ratio, *DIS* Difficulty initiating sleep, *DMS* Difficulty maintaining sleep, *EMA* Early morning awakening

* *p* < 0.05, ** *p* < 0.001

^a^ aOR indicated OR adjusted for all other explanatory variables

**Table 3:** Logistic regression model for associated factors with absence

	Blue-collar (*n* = 7292)		White-collar (*n* = 10,536)	
	Ref. None (*n* = 6690) / ≥ 1 day (*n* = 602)		Ref. None (*n* = 9473) / ≥ 1 day (*n* = 1063)	
Explanatory variables	Crude OR (95% CI)	aOR^a^ (95% CI)	Crude OR (95% CI)	aOR^a^ (95% CI)
Age	1.02 (1.01–1.03)^**^	1.02 (1.01–1.03)^**^	1.01 (1.00–1.01)	1.01 (1.00–1.02)
Sex				
Male	1.0	1.0	1.0	1.0
Female	1.52 (0.83–2.59)	1.35 (0.73–2.33)	1.47 (1.26–1.71)^**^	1.34 (1.13–1.58)^*^
Living alone				
No	1.0	1.0	1.0	1.0
Yes	0.84 (0.69–1.01)	0.86 (0.70–1.04)	1.16 (0.99–1.35)	1.08 (0.92–1.26)
Working pattern				
Daytime worker	1.0	1.0	1.0	1.0
Shift worker	0.95 (0.74–1.21)	0.92 (0.70–1.20)	0.98 (0.78–1.20)	0.46 (0.07–1.55)
Working time				
40–60 h	1.0	1.0	1.0	1.0
< 40 h	1.18 (0.91–1.51)	1.04 (0.80–1.35)	1.26 (1.09–1.46)^*^	1.15 (0.98–1.35)
≥ 60 h	0.68 (0.54–0.85)^*^	0.77 (0.60–0.97)^*^	0.93 (0.70–1.22)	0.93 (0.68–1.25)
Physical health				
Good/average	1.0	1.0	1.0	1.0
Poor	2.74 (2.24–3.33)^**^	2.85 (2.25–3.58)^**^	3.99 (3.45–4.62)^**^	3.60 (3.04–4.25)^**^
Mental health				
Good/average	1.0	1.0	1.0	1.0
Poor	1.55 (1.23–1.93)^**^	0.96 (0.73–1.25)	2.25 (1.90–2.65)^**^	1.08 (0.88–1.31)
Sleep duration				
7–8 h	1.0	1.0	1.0	1.0
< 7 h	0.86 (0.71–1.05)	0.90 (0.74–1.10)	0.91 (0.79–1.07)	0.87 (0.75–1.02)
≥ 8 h	1.38 (1.02–1.84)^*^	1.30 (0.96–1.74)	1.36 (1.04–1.76)^*^	1.25 (0.95–1.64)
Insomnia symptoms				
Without DIS	1.0	1.0	1.0	1.0
Presence of DIS	1.76 (1.35–2.27)^**^	1.56 (1.17–2.05)^*^	1.77 (1.45–2.15)^**^	1.27 (1.02–1.56)^*^
Without DMS	1.0	1.0	1.0	1.0
Presence of DMS	1.44 (1.16–1.76)^*^	1.15 (0.91–1.45)	1.84 (1.58–2.13)^**^	1.42 (1.20–1.67)^**^
Without EMA	1.0	1.0	1.0	1.0
Presence of EMA	1.04 (0.81–1.32)	0.79 (0.60–1.03)	1.32 (1.11–1.56)^*^	0.95 (0.78–1.14)
Hosmer-Lemeshow goodness of fit test		*p* = 0.543		*p* = 0.156

*OR* Odds ratio, *CI* Confidence interval, *aOR* Adjusted odds ratio, *DIS* Difficulty initiating sleep, *DMS* Difficulty maintaining sleep, *EMA* Early morning awakening

* *p* < 0.05, ** *p* < 0.001

^a^ aOR indicated OR adjusted for all other explanatory variables

## Discussion

This is the first study to investigate the difference in the association of insomnia symptoms and work productivity between the blue-collar and white-collar workers engaged in construction/civil engineering work.

In the present study, poor work performance was more prevalent among blue-collar workers. This result was consistent with a previous report in which presenteeism was more severe in plant workers than in office workers [4]. On the other hand, absence was more prevalent among white-collar workers. The percentages of workers with insomnia symptoms were similar between blue-collar and white-collar workers. Work performance was also associated with the respective insomnia symptoms among both blue-collar and white-collar workers. These results suggest that deteriorated work performance is susceptible to any kind of insomnia symptom in both job types. On the other hand, the association between absence and insomnia symptoms differed between them. Among blue-collar workers, absence was associated with DIS, but not with DMS or EMA. In addition to DIS, DMS was also associated with the absence of white-collar workers. These results suggested that DIS was strongly associated with the absence.

Interestingly, our study revealed a significant association between engagement in shift work and poor work performance among blue-collar workers. It is well known that about 20–30% of shift workers experience night time insomnia symptoms and daytime sleepiness, which are the main accepted symptoms of shift work disorder [16–18]. However, in the present study, the association between being engaged in shift work and poor work performance was shown independent of insomnia symptoms. The reason for this phenomenon is unclear; however, circadian misalignment-related symptoms (e.g., fatigue feeling, excessive daytime sleepiness, poor concentration, and mood changes) [19], possibly contributed to a loss in work performance.

In contrast to previous studies that reported a U-shaped association between sleep duration and work productivity [20, 21], the present study showed no association of short sleep duration with work productivity, while long sleep duration was associated with poor work performance. The U-shaped association of sleep duration was not confirmed in the present study due to difference in sleep duration among target population between previous studies and our study. The percentage of workers with short sleep duration (< 7 h) were 11–37% in previous studies [20, 21], in contrast to approximately 70% in our study, which is consistent with the reported rate (67%) among Japanese general population in the statistics survey conducted by Ministry of Health, Labour and Welfare [22]. Thus, the high percentage of short sleep duration in the present study might mask the formation of a U-shaped association between sleep duration and decreased work productivity.

This study had some limitations. First, insomnia symptoms were defined using a self-administered questionnaire and did not necessarily represent physicians’ diagnoses. Other sleep disorders, including sleep apnea, were not considered in the present study. Thus, a differential diagnosis of insomnia and other sleep disorders could not be made in the present study. In addition, the duration of insomnia morbidity and severity of the respective insomnia symptoms could not be estimated. The survey was conducted using a self-administered questionnaire, the repeatability and reliability of which were not assessed. Secondly, the causal relationship between work productivity and related factors could not be ascertained in this cross-sectional study. In addition, the differences in the causes of deteriorated work productivity and insomnia symptoms could not be estimated in the present study. For example, poor work performance and fewer absences among blue-collar workers could have been caused by job-related factors, including allowance for sick pay and sick days. Meanwhile, insomnia symptoms could have been associated with physical activity. However, the results obtained in this study cannot be used to examine these possibilities. According to a 2009 Estonia investigation of the impact of sick-pay cuts—initiated in response to an economic crisis—the percentage of blue-collar workers with low salaries who were on sick leave was lower than that of white-collar workers. These results suggest that lower-income was a major factor hindering them from using their sick-leave days [23]. Future studies should evaluate job-related factors, including the allowance for sick pay or sick days. Third, our study sample may not be a representative of Japanese general workers. However, the present study was thought to provide significant findings in blue-collar and white-collar workers engaged in construction/civil engineering work. Further studies with prospective sleep evaluation and therapeutic interventions would be desirable. Furthermore, we must clarify the relationship between the job description and the meaning of work performance among blue-collar workers.

## Conclusions

The present study revealed the association between insomnia symptoms and work productivity, suggesting the necessity of early prevention of insomnia among workers. In addition, the present study provided significant findings in both blue-collar and white-collar workers engaged in construction/civil engineering work. These findings about the prevalence of insomnia and its symptoms, the accrual situation of work productivity, and its associated factors among construction/civil engineering workers, could cause economic loss as well as possible accidents at the building site. However, further studies with prospective sleep evaluation and therapeutic interventions are needed.

## Supplementary Information

**Additional file 1. **STROBE Statement—Checklist of items that should be included in reports of *cross-sectional studies.*

## Data Availability

The datasets used and/or analyzed during the current study are available from the corresponding author on reasonable request.

## Abbreviations

EMA: Early morning awakening
DIS: Difficulty initiating sleep
DMS: Difficulty maintaining sleep
